# Artificial Neural Networks in Evaluation and Optimization of Modified Release Solid Dosage Forms

**DOI:** 10.3390/pharmaceutics4040531

**Published:** 2012-10-18

**Authors:** Svetlana Ibrić, Jelena Djuriš, Jelena Parojčić, Zorica Djurić

**Affiliations:** Department of Pharmaceutical Technology, Faculty of Pharmacy, Belgrade University, Vojvode Stepe 450, Belgrade 11221, Serbia; Email: jelena.parojcic@pharmacy.bg.ac.rs (J.D.); pjelena@gmail.com (J.P.); zorica@pharmacy.bg.ac.rs (Z.D.)

**Keywords:** artificial neural networks, modified release, pharmaceutical development

## Abstract

Implementation of the Quality by Design (QbD) approach in pharmaceutical development has compelled researchers in the pharmaceutical industry to employ Design of Experiments (DoE) as a statistical tool, in product development. Among all DoE techniques, response surface methodology (RSM) is the one most frequently used. Progress of computer science has had an impact on pharmaceutical development as well. Simultaneous with the implementation of statistical methods, machine learning tools took an important place in drug formulation. Twenty years ago, the first papers describing application of artificial neural networks in optimization of modified release products appeared. Since then, a lot of work has been done towards implementation of new techniques, especially Artificial Neural Networks (ANN) in modeling of production, drug release and drug stability of modified release solid dosage forms. The aim of this paper is to review artificial neural networks in evaluation and optimization of modified release solid dosage forms.

## 1. Introduction

The recent regulations from federal agencies, to apply the Quality by Design approach in pharmaceutical development, have compelled researchers in pharmaceutical industry to employ design of experiments (DoE) during product development. The benefits of DoE in product and process optimization are evident; it is the most convenient way to link the drug and excipient attributes and the process parameters to the Critical Quality Attributes (CQAs). Furthermore, its usage is necessary in defining and controlling Design Space [1]. Design Space is the multidimensional combination and interaction of input variables (e.g., material attributes) and process parameters that have been demonstrated to provide assurance of quality.

In the 1980s the use of DoE, especially factorial design, was generalized in the development of solid dosage forms. Statistical tools allowed the determination of critical process parameters of complex processes, as well as improvement and optimization of formulations [2].

Among diversity of DoE techniques, Response Surface Methodology (RSM) is the most frequently used optimization technique [2], which allows using polynomial equation(s) to link monitored responses to inputs:

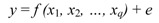
(1)
where *y* is response and *x*_1_, *x*_2_, …, *x_q_* are inputs. 

This equation presents a mathematical description of a relationship between inputs and output. The advantage of this empirical model is that one can easily, without knowing true (*i.e.*, mechanistic) link between inputs and outputs, describe their relationship. However, since the relationship between inputs and outputs is often more complicated, the observed model sometimes results in poor estimation of optimal conditions [2]. Drawbacks of this statistical approach were overcome in the 1990s, when, with the development of computer science, researchers started to apply machine learning.

Machine learning is a branch of artificial intelligence. It is a scientific discipline involved in design and development of algorithms that allow computers to evolve behaviors based on empirical data. Machines “learn” to automatically recognize complex patterns, to distinguish between exemplars based on their different patterns, and to make intelligent decisions. There are several machine learning disciplines, such as: decision tree learning, association rule learning, artificial neural networks, genetic programming, support vector machines, clustering, Bayesian networks, *etc*. [3]. Among these, artificial neural networks are the most frequently applied in pharmaceutical development [4]. 

In order to define and control the design space, the most effective approach is to complement DoE with some of the more advanced machine learning techniques, such as ANNs. DoE allows easy set-up of levels and boundaries of the materials attributes and processing parameters that are influencing CQAs. But, the linkage between the input(s) and the output(s) often requires non-linear modeling techniques that are advanced in comparison to DoE approaches. DoE models are polynomial, whereas machine learning offers a diverse variety of classification and modeling techniques. 

## 2. Artificial Neural Networks

Artificial neural networks (ANNs) are computer programs, which recognize patterns in a given collection of data and produce model for that data. It emulates brain in two respects: (1) knowledge is acquired by the network through a learning process (trial and error); and (2) interneuron connection strengths (*i.e.*, synaptic weights), are used to store the knowledge [4,5].

Because neuroscience is still full of questions and because there are many levels of abstraction and many ways to take inspiration from the brain, there is no single definition of what an artificial network is. Most would agree that it involves a network of single processing elements which can exhibit complex behavior determined by the connections between the processing elements and element parameters [4,5].

Since design of artificial networks has been inspired by the structure of the human brain, it is pertinent to review/compare the function of biological neural network *versus* artificial ones.

**Figure 1:**
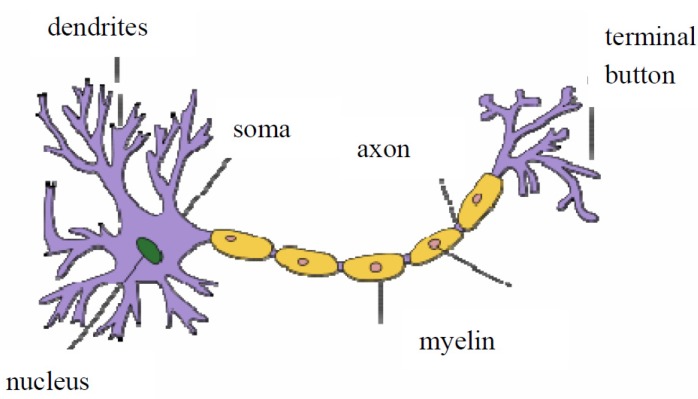
Schematic representation of biological neuron.

The fundamental processing unit of the human brain is the neuron (Figure 1). The neurons are linked together by dendrites, which serve to deliver messages to the neuron. Each neuron has an output channel, known as an axon by which signals can be transmitted unchanged or altered by synapses. 

**Figure 2:**
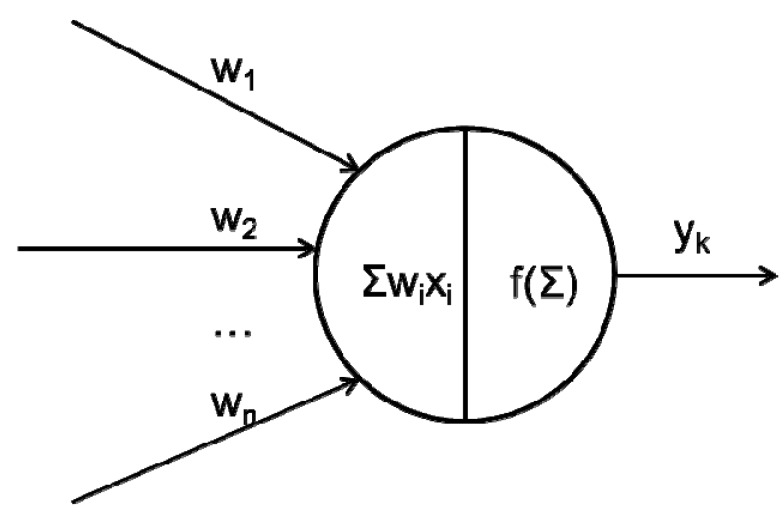
Schematic representation of artificial neuron.

In the artificial neural network the logic processing unit is the neuron (“neurode”, “processing element” (PE) or “unit”), which takes one or more inputs and produces an output. At each neuron every input has an associate weight (*w*_1,_...*w_n_*, Figure 2) that defines relative importance of each input connected to the neuron. Set of inputs are multiplied with sets of weights; those set of inputs are summed together, and fed into a function to produce an output (*y_k_*, Figure 2).

Every ANN is defined with architecture (number of layers, in each layer number of neurons, way in which neurons are connected, inter-neuron connections) and learning algorithm [4].

### 2.1. What Happens between Neurons in a Network?

**Figure 3:**
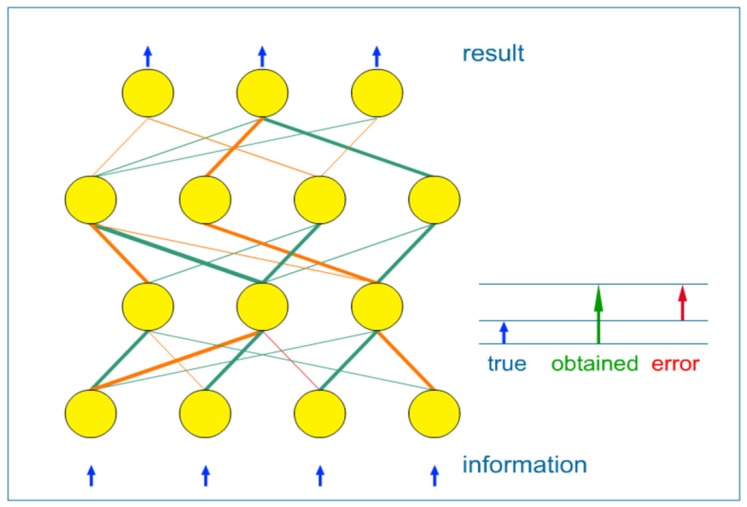
Schematic representation of feed-forward neural network.

In Figure 3, the simplest ANN is presented, which is a feed-forward neural network. A set of system inputs are transferred to the other neurons through synapses. The synapses store parameters called “weights” that manipulate the data in the calculations. Therefore, the set of inputs are multiplied with a set of adjustable weights. Weights are fed into the set of processing elements which then produce the set of outputs. The set of outputs are fed in another layer of weights, following another layer of processing elements, till the last processing layer, which produces output of the neural network.

ANN is defined by the following parameters: (1) interconnection pattern between different layers of neurons; (2) the learning process for updating the weights of the interconnections; and (3) activation function that convert a neurons input to its output activation. Activation functions that are used in ANNs are sigmoidal, piecewise linear, step, Gaussian, exponential, logistic sigmoidal, *etc*. [3].

Training of the network is in fact the “learning process”. It could be divided into:

(1) *supervised learning*: when network weights are adjusted using the known data, split into input-output pairs. Supervised learning is the most commonly used training method for ANNs and will be further explained in this paper;
(2) *unsupervised learning*: it refers to the problem of trying to find hidden structure in unlabeled data. Since the examples given to the learner are unlabeled, there is no error or reward signal to evaluate a potential solution;
(3) *reinforcement learning*: which differs from standard *supervised learning* in that correct input/output pairs are never presented.

Training of an ANN is actually adjustment of weight values in order to obtain the best nonlinear relationship between parameters used as inputs and outputs of the network. At the beginning of the training process, weights between the neurons have random values. During the training phase input/output data pairs are presented to the network, and the network searches for the input-output relationships. One cycle of input-output presentation to the network is called an iteration (epoch). Process of the network training can be thought of as search for the optimal weights values that successfully convert inputs to outputs through sometimes numerous iterations. This process is often called convergence [5]. During the process of the weights adjustment (*i.e.*, network training), some of the interconnections are strengthened and some are weakened, so that a neural network will output a more correct answer [4]. Once the optimal set of weight values is found, the network training stops and squared error between the actual (training) output values and the ones predicted by the network is minimal at this point. At the beginning of the training process, data is divided in two subsets: training and test. Training data is used to search for the optimal weight values, whereas by using test data the network checks its predictive ability internally. The sum of squared errors (SSE) for the training and test subsets can be calculated using the following equation [6]:

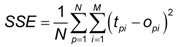
(2)
where *o_pi_* is the output predicted by the network of *i*th output node from *p*th sample; *t_pi_* is the training (actual) output of the *i*th output node from *p*th sample; *N* is the number of the training samples and *M* is the number of the output nodes. 

In the process of the network training, the progress of change of error for both training and test data sets are evaluated simultaneously. In the case of training data set, *SSE* decreases indefinitely with increasing number of hidden nodes or training iterations (epochs) [5]. Initially, *SSE* for the training set decreases rapidly due to learning, whereas its subsequent slower decrease is attributed to memorization or overfitting (if the number of training cycles or hidden nodes is too large). In the case of test data set, *SSE* decreases initially but subsequently increases due to memorization and overfitting of the ANN model [5]. It is therefore recommended to stop the training once the test error starts to increase and to select the number of hidden nodes when the test error is minimal [5]. Steps in the supervised network training and usage are presented in Table 1.

**Table 1:** Steps in the supervised network training and usage.

***Training of the network***
Data is presented to the network Network computes an output Network output is compared to desired output Network weights are modified to reduce error
***Usage of the network***
Present new, unseen data to the network Network computes an output based on its training

It is important to emphasize that ANNs work as “black boxes”. It is not possible to see the mathematical relationship between inputs and the output. 

### 2.2. Basic Topologies (Architectures) of Artificial Neural Networks

Generally, ANNs could be divided into static ANNs and dynamic ANNs. Differences between static and dynamic networks are in the way in which signals are transmitted through the network.

In order to obtain relevant and reliable results by using ANN models, it is recommended that the number of experimental runs is 10 times greater in comparison to the number of inputs; or, if this is not feasible, at least two to three times the number of inputs [5]. It is, therefore, practical to first conduct screening experimental design, in order to select the most significant input variables that influence output properties since it leads to reduction of the number of the ANN inputs (and number of the examples needed for the network training at the same time). 

#### 2.2.1. Static Networks

There are numerous types of static neural networks. The most frequently used static neural network in pharmaceutical development is the multilayer perceptron network.

**Figure 4:**
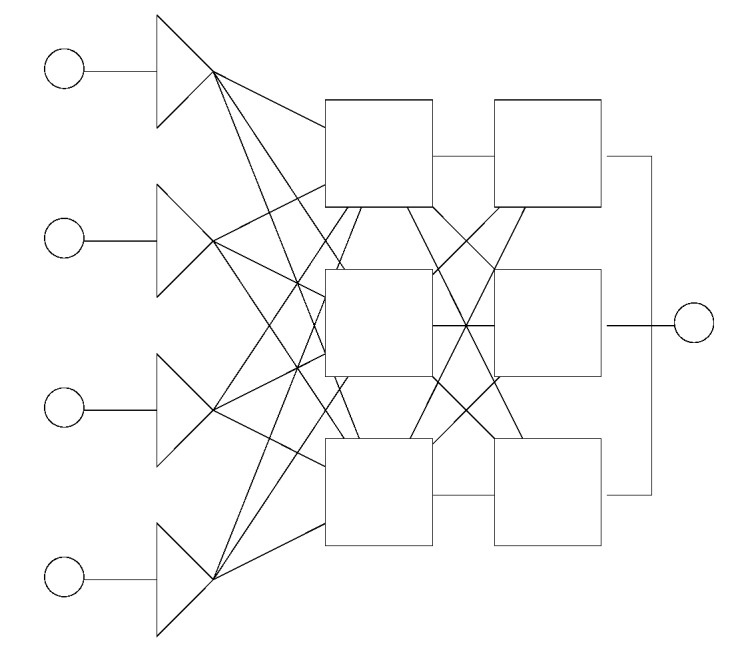
Example of Multilayer perceptron (MLP) network architecture with four layers (first layer with four neurons, second and third layers with three neurons and fourth layer with one neuron).

Multilayer perceptron (MLP) is the simplest neural network architecture, that is a *feedforward artificial neural network* model that maps sets of input data onto a set of appropriate outputs. An MLP consists of multiple layers of nodes in a directed graph, with each layer fully connected to the next one (Figure 4). Except for the input neurons, each neuron has a nonlinear activation function. MLP utilizes a *supervised learning* technique called the *backpropagation* algorithm [7,8]. For an MLP neural network, the relationship between the inputs and outputs can be represented as follows:

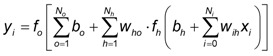
(3)
where *x_i_* and *y_i_* are networks primary input and output; *w_ih_* and *w_ho_* (*i* = 1, 2, …, *N_i_*; *o* = 1, 2, …, *N_o_*) are the weights of the connections between the input and hidden units, and between the hidden and the output units, respectively. *b_h_* and *b_o_* are biases of hidden units and output units, and *f_h_*(*·*)and *f_o_*(*·*)are hidden and output functions respectively [9]. Biases are neurons that always have constant values and serve to allow certain shifting in the predicted outputs (*i.e.*, they can be thought of as function intercepts). 

**Figure 5:**
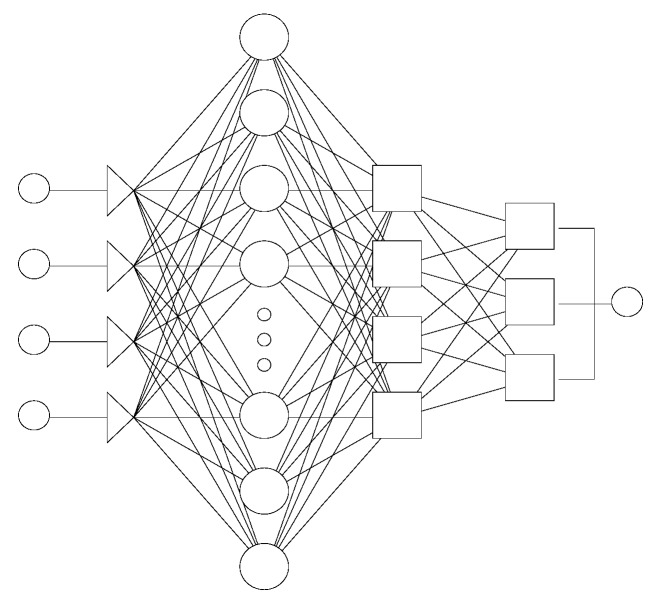
Example of generalized regression neural network (GRNN) architecture.

Generalized regression neural network (GRNN) was first introduced by Specht [10]. It models the function directly from the data. It was recognized that GRNNs have been derived from statistical method of function approximation [11]. GRNNs always have exactly four layers: input layer, a layer of so-called radial centers, a layer of regression units and output layer (Figure 5). 

Radial layer is used to perform clustering on the known, training data. Clustering algorithms usually used for training of this layer are sub-sampling, K-means or Kohonen [12]. The number of clusters, *i.e.*, radial units (neurons) can be optimized and usually it corresponds to the number of the training samples. Regression layer has one unit (neuron) more than the output layer and their activation function is linear. One extra unit (in comparison to the output layer) is used for calculation of the probability density whereas remaining units are used for calculation of outputs. In the output layer a specialized function is applied where the calculated outputs of the regression layer are divided by the probability density [12].

Fundamental equation of GRNN can be presented as:

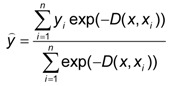
(4)
where the distance *D* between the training sample and the point of prediction, is used as a measure of how well the each training sample can represents the position of prediction, using the standard deviation or the smoothness parameter, *σ* [10].

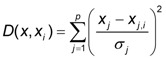
(5)

The advantage of the GRNN is that its training is much faster compared to MLPs.

#### 2.2.2. Dynamic Neural Networks

Dynamic (or recurrent) networks have the ability to store data and elaborate it in time. That is the advantage of dynamic neural networks (compared to the static networks). Every input is analyzed as a function of the previous input. The network remembers past inputs; therefore, the current output is integration of past inputs and current response of the system [13].

Connections between the neurons can be set up to have a memory which is very important for dynamic networks. The order of the memory says how many time steps the signal will be delayed. Treatment of dynamic data requires this kind of temporal dependencies of signal channeling. Network topology (architecture), together with the control system for time delay of the signal, forms a complete system. Correction of weights in the dynamic neural network is somewhat more complicated, in comparison to static neural networks. It is possible to use the technique called backpropagation through time where the backpropagated signal is buffered and reversed which enables getting forward and backpropagated signals synchronized in time [13]. 

Gamma memory is a specific short memory recurrent structure which preserves temporal information about the system. Distinctive feature of gamma memory is the number of taps-number of signal delays. For a given number of taps memory remembers previous system states and integrates them with current ones. From the point of view of signal transmittance Gamma memory can be seen as recursive low-pass filter (each output gives a more filtered version of the original signal) which acts as an infinite response filter. It is ideal for adaptive systems since its interpolation weight *μ* can be adapted using the usual algorithms. An important class of memory filters is based on the (non-dispersive) delay operator. Gamma memory is actually a combination of Tapped-Delay-Line (TDL) and a simple feedback neuron. So, the signals *g_k_*(*t*) at the taps *k* in time *t* of the Gamma memory are convulsions of the impulse response *g_k_* tap *k* [13]*.*

A *K*-th order tapped delay line, the memory structure for Time Delay Neural Network (TDNN) [14], can be regarded as a single-input-*K*-output filter with impulse responses *gk*(*t*) = d(*t* − *k*). 

Depending on the way that signals are sent back in the neural network, partial or full recurrence exists. Partial recurrence occurs, for example, when recurrent connections are sent from the hidden layer back to itself. In the fully recurrent networks the final output (of the network) is sent back into the network. If connections between the neurons are set up to have a memory then the order of the memory says how many time steps the signal will be delayed (default value is one time step, *i.e.*, −1). Over time, the network stores long term memory structure in its feedback (recurrent) and regular connections, whose weights are adjusted during the training [15].

Elman neural network (ENN) is considered as a special kind of feed-forward networks that has additional memory neurons and local feedback [16]; therefore it is a simple dynamic neural network. Recurrent links are used to provide network with a dynamic memory when hidden unit patterns are fed back to themselves [17]. Activation functions of neurons in the hidden layer are fed back to copied layer at every time step to provide an additional input in conjunction with other input features. This recurrence gives the network dynamic properties, which make it possible for the network to have internal memory [18]. 

**Figure 6:**
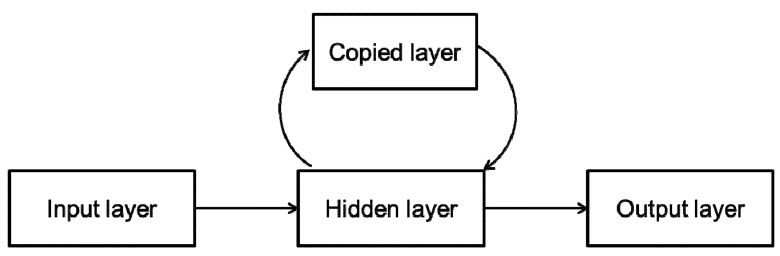
Topology of Elman neural network.

Essential characteristic of Elman neural network (Figure 6) is the fact that a copied (context) layer exists that serves as the temporary memory of the hidden layer outputs. Once the signal is transferred from the input layer to the hidden layer, it is elaborated and sent both to output and copied layer. During the first input–output cycle, copied layer is empty, *i.e.*, its outputs are equal to 0. Then, when the activation function in the hidden layer is applied for the first time its outcomes are sent to output and copied layer. During the second training cycle, the hidden layer receives inputs from both input and copied layer which are then combined and analyzed simultaneously. The process is repeated for the next training cycles so that each time the copied layer contains values of the outputs of hidden layer from the previous training cycle. This type of signal recurrence is recognized as a one-step time delay [9]. The algorithm of ENN can be represented as follows:

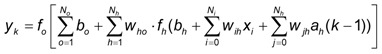
(6)
where *x_k_* and *y_k_* are networks primary input and output; *w_ih_*, *w_jh_* and *w_ho_* (*i* = 1, 2, …, *N_i_*; *j*,* h* = 1, 2, …, *N_h_*; *o* = 1, 2, …, *N_o_*) are the weights of the connections between the input and hidden units, between the copied and the hidden units and between the hidden and the output units, respectively. *b_h_* and *b_o_* are biases of hidden units and output units, and *f_h_*(*·*)and *f_o_*(*·*)are hidden and output functions respectively. The weights of the copied layer determine the impact of signal recurrence on the networks output. 

### 2.3. Application of ANNs in Formulation and Evaluation of Modified Release Dosage Forms

A pioneer work in the field was a paper published by Hussein *et al*. in 1991. They modeled the *in vitro* release characteristics of drugs in matrices prepared from various hydrophilic polymers [19,20]. Neural networks with a single hidden layer were applied to predict drug release from matrix tablets. Predicted drug release profiles were comparable with those generated through the use of statistical analysis [19].

Chen and coworkers [21] studied the influence of seven formulation variables and three tablet variables (moisture, particle size and hardness) on dissolution-time profiles at ten sampling times. Twenty two formulations were used for the network training. The optimized ANN model was constructed by selecting the optimal number of hidden layers and neuron number in each layer. Optimized ANN model was used for prediction of formulations based on two desired target dissolution profiles, as well as two desired bioavailability profiles.

In modeling of a roller-compaction process, Turkoglu *et al.* [22] varied four formulation and process variables through 42 experiments. Results were analyzed using ANN with genetic algorithms. When a set of validation experiments was analyzed, genetic algorithm predictions of tablet characteristics were much better than the ANN. 

Optimization of theophylline release from controlled release tablets was performed according to desirable drug release profiles, based on the plasma concentration profiles of theophylline predicted by the pharmacokinetic analysis in humans [23]. Experimentally observed drug release profiles from 16 formulations were used for ANN training. Simultaneous optimization was performed by minimizing the generalized distance between the predicted values of each response and the desirable one that was optimized individually. Similar study was performed by Peh *et al*. [24] with optimization of glyceryl monostearate matrix tablets. MLP neural network was trained and the closeness between the predicted and the reference dissolution profiles was investigated using similarity factor (*f*2).

In a number of papers, ability of ANNs to predict controlled release profiles of a drug was compared with the response surface methodology [25,26]. In general, formulations were optimized using both RSM and ANNs. Afterwards, predicted profiles were compared with experimentally observed. Differences between predicted and experimentally observed results were discussed. 

Generalized regression neural network was applied in optimization of formulation of aspirin matrix tablets with Eudragit RS and Eudragit L, as matrix forming materials [12,27]. The percent of Eudragit and tablet hardness were inputs of the network, varied according to the central composite design, while percent of aspirin released at 4 sampling time points, as well as release exponent of Korsmayer–Peppas equation were outputs of the network. In another study, the same authors applied GRNN network in predicting drug stability in matrix tablets [28]. Optimization of formulation with respect to drug release profile, as well prediction of drug stability showed abilities of GRNN network in formulation of controlled release preparations. It seems that this type of network has ability to optimize controlled release formulations better then MLP network. Also, training with GRNN network is much faster than with other network types [12]. 

**Figure 7:**
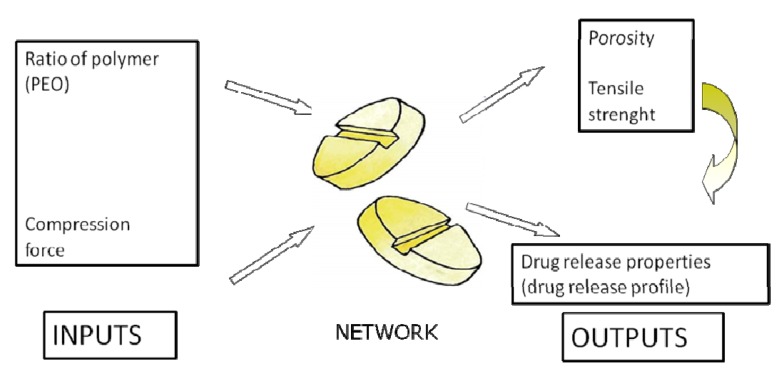
Schematic representation of inputs and outputs for the optimization of matrix tablets [30].

Dynamic neural networks were for the first time applied in the prediction of drug release profiles from controlled release formulation in 2002 [18]. Drug release profiles were treated as time series curves in which information contained in one time point affects further predictions. Elman recurrent network was applied. Since in dynamic neural networks data is stored and elaborated in time, it is expected that it could be useful in time dependent processes, like drug release prediction or drug stability issues. Therefore, our research group have successfully applied dynamic neural networks in establishing design space for formulation of matrix tablets and compared those results with static networks [13,29,30]. Following case study presents our unpublished data and could be useful for the readers as a guide in selection and development of appropriate network for the problem studied. The aim of the study was to develop an ANN model for optimization of matrix tablets mechanical properties and drug release profile as well. Inputs were ratio of matrix forming material (Polyox WSR 1105 and Polyox WSR Coagulant) and compression force, while mechanical properties (porosity and the tensile strength) of tablets, as well as drug release profiles were outputs of the network (Figure 7). 

**Figure 8:**
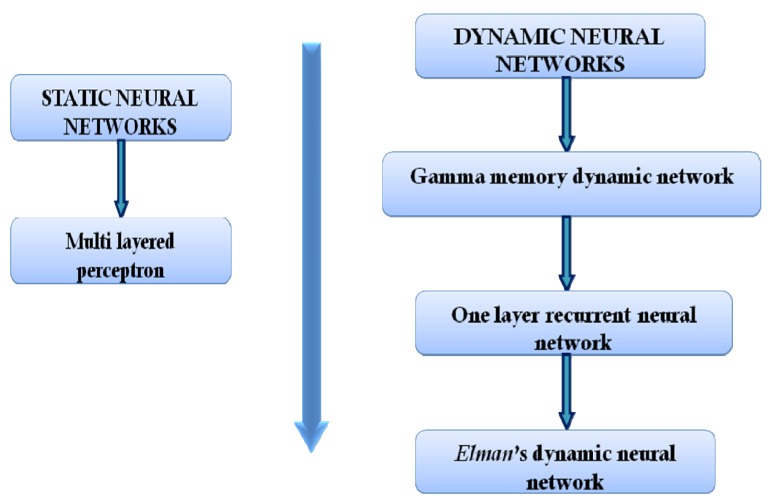
Flow diagram for the network architecture selection [30].

Eleven formulations were prepared (nine, according to full 3^2^ factorial design for network training and two for network testing). Network topology was chosen according to the flow diagram presented in Figure 8. 

**Figure 9:**
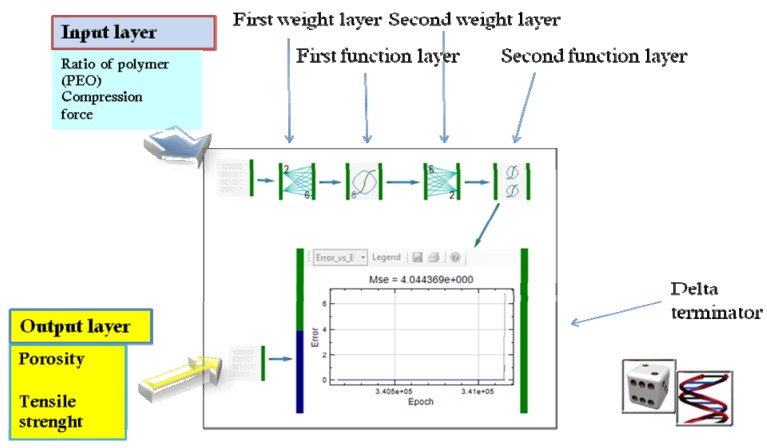
Schematic representation of MLP network.

Firstly, MLP network was chosen, as the most frequently used static neural network. The structure of MLP network used in the study is presented in Figure 9. 

When the trained MLP network was tested using the test formulations with respect to mechanical properties, the model developed by MLP network enables prediction of porosity and tensile strength for test formulation, on the basis of known compression force and weight ratio of PEO polymer used (Table 2). 

**Table 2:** Observed *versus* MLP predicted values for test formulations.

Formulation	Observed values Porosity (%) and tensile strength (MPa)	Predicted values Porosity (%) and tensile strength (MPa) (*r*^2^ = 0.9982)
Test 1	19.55 ± 0.49 1.304 ± 0.042	20.34 ± 0.78 1.313 ± 0.155
Test 2	17.55 ± 0.55 1.661 ± 0.035	17.33 ± 0.78 1.539 ± 0.155

**Figure 10:**
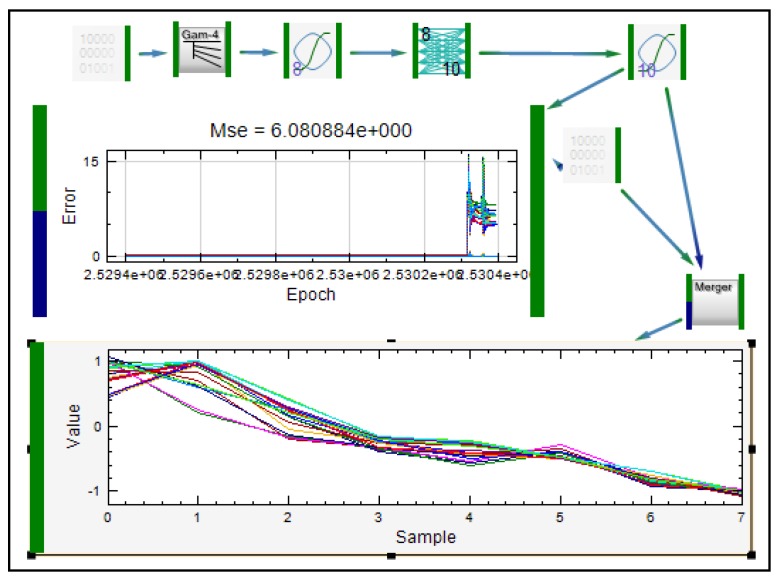
Schematic representation of gamma memory dynamic neural network (GMDNN).

Afterwards, the same MLP network was used for training, when the outputs were drug release profiles. Percents of drug released in predetermined time intervals were outputs of the network. Inputs of the network were the same as in previous case. After the training session, the established network was not able to predict drug release profiles for test formulations. The calculated values for similarity factor indicated that profiles are not similar (*f*2 55.37 and 48.22 for test 1 and test 2 formulation, respectively). The next step was to change the network type and to evaluate the drug release prediction using dynamic neural networks. Gamma memory one layer dynamic neural network was applied (GMDNN) (Figure 10). Gamma memory (GM) used had 4 taps. 

**Figure 11:**
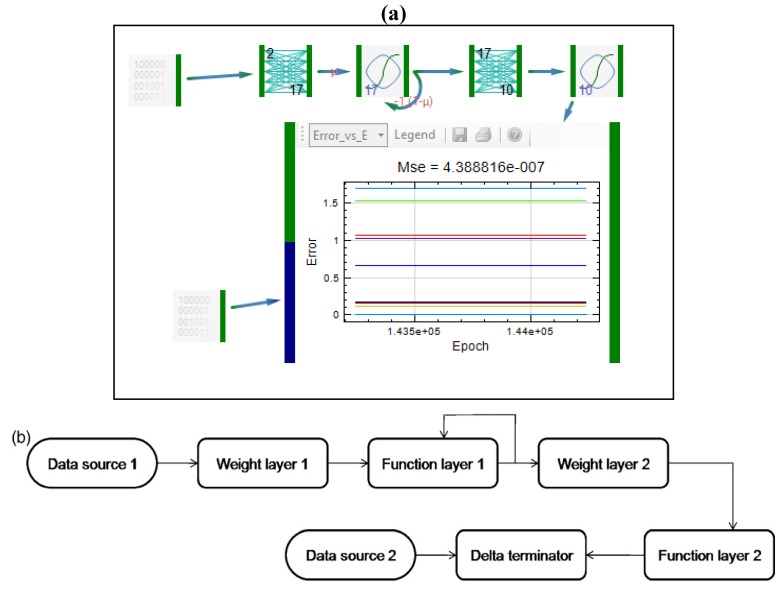
Representation of recurrent one layer dynamic neural network (OLDNN): (**a**) Peltarion^®^ software layout and (**b**) network’s schematic representation.

From GM signal is transferred to the first function layer, then to the weight layer and subsequently to the second function layer. Delta terminator compares two signals: one comes from the data source and represents real, observed outputs; whereas the second signal comes from the second function layer and represents outputs predicted by the dynamic system. The number of inputs and outputs in weight and function layers, as well as tapped delay line, were optimized using Monte Carlo simulations. As in the case of model developed by MLP, the obtained model does not predict drug release successfully. GM predicted shifting of profiles towards higher percentage of drug released after second hour of study, which was not confirmed experimentally. Topology of the recurrent one layer dynamic neural network (OLDNN), which was next used in the training, is presented in Figure 11. 

**Figure 12:**
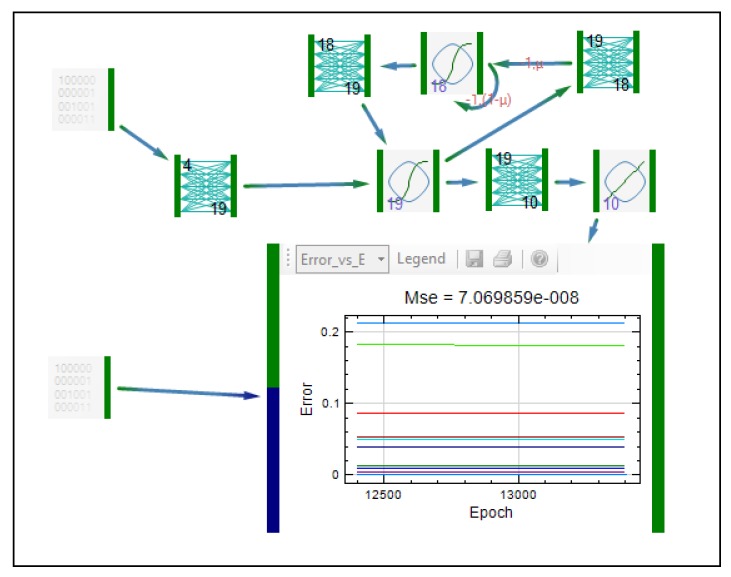
Representation of Elman dynamic neural network.

Most of the networks elements were the same as in previously described GMDNN. From the first data source inputs are transferred to the first weight layer. The first weight layer has 2 inputs and 17 outputs. The number of its outputs was optimized using Monte Carlo simulations. OLDNN is a partially recurrent network since the recurrent connections are sent from the hidden layer back to itself (first function layer). In the fully recurrent networks the output is sent back into the network. From the first, recurrent function layer, signal goes to the second weight layer that has 17 inputs and 10 outputs. Then the signal goes to the second functional layer, which is an ordinary function layer. Once again, the network was trained and afterwards tested. The model obtained using OLDNN enabled adequate prediction of drug release profiles, with similarity factors for test formulations 64.01 and 77.54, respectively. Even though results obtained using this network were better, there were still shifting values of predicted drug release profiles compared to experimentally observed values at later time points. Therefore, in the next study [29], the starting point of experiments was changed: since it was demonstrated that mechanical properties of the tablets have impact on drug release, porosity and the tensile strengths were selected to be inputs, together with ratio of the polymer and compression force. Elman dynamic neural network was applied, with topology shown in Figure 12. This type of network was also applied in modeling of drug release from lipid matrix tablets [29]. After the training session, prediction of drug release from test formulations was better in comparison to OLDNN application. 

In Table 3, studies on formulation, characterization and optimization of modified release formulations are listed, with respect to inputs/outputs selected, type of the network used, and the authors and year of performed studies.

**Table 3:** Formulation, characterization and optimization of modified release solid dosage forms are listed, with respect to inputs/outputs selected, type of the network used, and the authors(year) of performed studies.

Formulation, characterization and optimization of modified release formulation		
Inputs/outputs/aim	Network type	Authors, year
Design of controlled release formulations. Varying formulation variables were used as inputs and *in vitro* cumulative percentages of drug released were used as ANN outputs.	MLP	Chen, 1999 [21]
Optimization of diclofenac sodium sustained release matrix tablets. Trained model was used to predict release profiles and to optimize the formulation composition.	MLP	Zupancic Bozic, 1997 [31]
Design of extended release aspirin tablets. The amount of Eudragit^®^ RS PO/Eudragit^®^ L-polymer and compression pressure were selected as inputs, whereas *in vitro* dissolution profiles, release order and release constant from the Korsmayer Peppas equation were selected as output parameters.	GRNN	Ibric, 2002, 2003, 2007 [12,27,28]
Prediction of drug dissolution profiles. Inputs for the network training were the matrix forming agents’ ratio, the time point of the measurement of percent dissolved, and the difference between the release rate of the preceding two time points of the predicted profile. *In vitro* dissolution profiles were used as network outputs.	MLP	Peh, 2000 [24]
Investigation of controlled drug release. Drug fraction and time were used as network inputs and *in vitro* dissolution profiles as outputs.	MLP	Reis, 2004 [32]
Prediction of dissolution profiles for matrix controlled release theophylline pellet preparation. Inputs for the network training were the matrix forming agents’ ratio, and the time point of the measurement of percent dissolved; *in vitro* dissolution profiles were used as outputs.	EDNN	Goh, 2002 [18]
Modeling of diclofenac sodium release from Carbopol 71G matrix tablets. Polymer and binder content were inputs, while *in vitro* dissolution profiles were outputs	MLP	Ivic, 2010a [26]
Modeling of diclofenac sodium release from polyethylene oxide matrix tablets. Polymer weight ratio and compression force were used as inputs, whereas *in vitro* dissolution profiles were used as networks outputs. Dissolution profiles were treated as time series using dynamic neural networks.	MLP, GMDNN, OLDNN	Petrović, 2009 [13]
Drug release control and system understanding of sucrose esters matrix tablets. Networks inputs were HLB values of sucrose esters (SEs), SEs concentration, tablet volume, tablet porosity and tablet tensile strength. *In vitro* dissolution profiles and parameters indicative of burst release mean dissolution time and release exponent were used as outputs.	MLP	Chansanroj, 2011 [33]
A number of unique ANN configurations are presented, that have been evaluated for their ability to determine an IVIVC from different formulations of the same product. *In vitro* dissolution data were used as inputs and associated outputs were pharmacokinetic time points from nine patients enrolled in a crossover study.	MLP, GRNN, RNN	Dowell, 1999 [34]
Development of level A *in vitro*–*in vivo* correlation. Inputs for the network training were *in vitro* dissolution samples whereas *in vivo* dissolution profiles calculated by numerical deconvolution for each volunteer individually were used as outputs.	GRNN	Parojčić, 2007 [35]
Prediction of relative lung bioavailability and clinical effect of salbutamol when delivered to healthy volunteers and asthmatic patients from dry powder inhalers (DPIs). Training of the ANN network was performed using *in vitro* aerodynamic characteristics of the formulation and demographic data of volunteers/patients as input parameters, whilst *in vivo* data (urinary excretion of the drug and its metabolite) were networks outputs.	MLP	De Matas, 2008 [36]
Prediction of kinetics of doxorubicin release from sulfopropyl dextran ion-exchange microspheres. Three independent variables, drug loading level, concentration of NaCl and CaCl_2_ in the release medium were used as the ANN inputs and the fractional release of doxorubicin at four different time points as the outputs.	MLP, HNN	Li, 2005 [37]
Prediction of drug release profiles in transdermal iontophoresis. Neural networks inputs were the process conditions of pH, ionic strength and current, as well as the time point. The output was the predicted permeation rate of the drug (diclofenac sodium).	RBFNN	Lim, 2003 [38]
Optimization of drug release from compressed multi unit particle system (MUPS) using generalized regression neural network (GRNN)	GRNN	Ivic, 2010b [39]

Abbreviations: MLP, Multilayered Perceptron; GRNN, Generalized Regression Neural Network; RBFNN, Radial Basis Function Neural Network; EDNN, Elman Dynamic Neural Network; GMDNN, Gamma memory Dynamic Neural Network; OLDNN, One Layer Dynamic Neural Network; HNN, Hierarchical Neural Network.

The mutual idea of presented studies could be described as follows: they attempted to create a relationship between formulation and/or process variables (**INPUTS**) in production of modified release preparation (regardless of whether they were matrix tablets or multiple unit systems) and drug release profiles or dissolution rate constants (**OUTPUS**). In the training session, prediction capability of the trained networks was tested comparing predicted and experimentally observed drug release profiles, using the similarity factor as parameter and/or correlation coefficient.

To date, there are many commercial, as well as freely available computer programs that can be used for ANN modeling. It should be pointed out that modeling of the same data through different software will give a little bit different results. An interesting study has been published by Chen and coworkers [40]. They compared four commercially ANN software programs to predict the *in vitro* dissolution profiles of controlled release tablets. The ANNs developed from the four software programs were validated by predicting the *in vitro* dissolution time-profiles of each of the 19 formulations, which were excluded from the training process. Although the same data set was used, the optimum ANN architectures generated from the four software programs was different. Nevertheless, it was concluded that the four programs provided reasonable predictions of *in vitro* dissolution profiles for the data set employed in this study.

## 3. Future Prospects

So far, a lot of work has been done concerning application of ANNs in optimizing drug release profiles from controlled release formulation. As neural computing is progressing, current trends and recently published articles [29,33,41,42,43] indicate that the future state in this area will include integration of ANNs with other machine learning techniques, such as fuzzy logic, genetic programming, decision trees, self-organizing maps, *etc*. Decision tree methodology, a nonparametric statistical technique, used for classification problems, could be successfully used in characterization of controlled release problems, as was reported. The main constraint of this methodology is that it requires a large data set, because inductive generalization given in the form of a decision tree is dependent on a sufficient amount of data [29]. 

There have been very few attempts to use genetic programming in the optimization of pharmaceutical formulations [42,43,44]. Published results indicate that symbolic regression via genetic programming is a promising technique; in comparison to ANN models, it provides parametric equations, which can be more easily interpreted, thus having a greater potential for understanding of the mechanisms of the modeled process [43]. 

However, ANN models are well established and could be used in implementation of Quality by Design concept, *i.e.*, understanding of Design Space and Quality Risk Management for modified release formulations. 
